# A proposed one-stop-shop approach for the delivery of integrated oral, mental, sexual and reproductive healthcare to adolescents in Nigeria

**DOI:** 10.11604/pamj.2020.37.172.22824

**Published:** 2020-10-21

**Authors:** Morenike Oluwatoyin Folayan, Nadia Adjoa Sam-Agudu, Abiola Adeniyi, Elizabeth Oziegbe, Nneka Maureen Chukwumah, Boladale Mapayi

**Affiliations:** 1 Department of Child Dental Health, Obafemi Awolowo University Teaching Hospitals Complex, Ile-Ife, Nigeria,; 2 International Research Center of Excellence, Institute of Human Virology Nigeria, Abuja, Nigeria,; 3 Division of Epidemiology and Prevention, Institute of Human Virology, University of Maryland School of Medicine, Baltimore, USA,; 4 Faculty of Dentistry, Lagos State University College of Medicine, Lagos, Nigeria,; 5 Department of Preventive Dentistry, College of Medical Sciences, University of Benin, Benin City, Edo, Nigeria,; 6 Department of Mental Health, Obafemi Awolowo University, Ile-Ife, Osun State, Nigeria

**Keywords:** Oral health, mental health, sexual and reproductive health, adolescent, integrated health service delivery, Nigeria

## Abstract

The interconnectedness of oral, mental, sexual, and reproductive health (OMSRH) in adolescents prompts exploration of novel approaches to facilitate comprehensive access of this population to the relevant health services. This paper proposes an integrated one-stop-shop approach to increasing adolescents' access to OMSRH care by leveraging on dental clinics as a template for integration, using a non-stigmatized platform to deliver stigmatized healthcare. Novel healthcare delivery models are needed to enhance adolescents' access to the comprehensive prevention and treatment services that they critically need. Effective, integrated health care for this population is lacking, especially across various health areas. This is a proposal for leveraging dental clinics for integrated OMSRH care, using facility-based services, to adolescents. Emphasis will be placed on reducing stigma as a barrier to service accessibility, acceptability, equitability and appropriateness. Empirical studies will be required to test the feasibility, validity and effectiveness of this proposed model.

## Perspectives

Adolescence is a life stage between childhood and adulthood, defined chronologically as ages 10 to 19 years. During this period, interrelated and overlapping physical, cognitive, psychological, moral, and socio-emotional development occurs, often rapidly. This development is affected by many factors: gender, peers, family, race, culture, community, and the environment [1]. Adolescents also foster connections to obtain peer approval, thereby increasing the risk of peer pressure [2]. The desire for connectedness with peers, evolving social and emotional maturity, and poor assessment of risk-benefit in life choices increase the likelihood of making decisions with negative consequences: substance abuse, early/teenage pregnancy, and sexually transmitted infections [2]. These forces have implications for oral, mental, sexual and reproductive health (OMSRH), which are linked [3]. Oral health is a state of being free from chronic mouth and facial pain, which may result from oral infection, cancers and periodontal diseases. These conditions may lead to tooth decay, tooth loss, and other diseases and disorders that limit a person´s biting, chewing, smiling, speaking, and psychosocial wellbeing. Oral health is a key indicator of overall health, wellbeing and quality of life. Poor oral health has implications for anyone coping with the stresses of life, work productively and community expectations, with implications for mental health well-being [4]. Good sexual and reproductive health (SRH) is also required for overall health and well-being as it is not merely the absence of disease, dysfunction or infirmity [5].

The connections between oral, mental, and sexual and reproductive health are multidimensional. Adolescents´ increasing independence as they emerge from childhood can lead to increased consumption of free sugar and suboptimal tooth brushing, with associated health problems. High intake of free sugar- a risk factor for caries- is associated with attention deficit hyperactivity disorder [6] and suicidal ideation [7] in adolescents. Suicidal tendency is linked to mental health problems that develop in adolescence from exposure to adverse life events and circumstances, such as chronic illness, being orphaned, being a member of minority or stigmatized groups, living in humanitarian and fragile settings, sexual violence and abuse, pregnancy, forced marriage, and parenthood and poverty. These circumstances tend to occur alongside innate psycho-biological risk factors that predispose to poor mental health [8]. Furthermore, mental health problems increase high-risk behaviors, including those that accentuate SRH risks and needs [9]. Adolescents, especially those in low- and middle-income countries (LMICs), have high unmet SRH needs in addition to high unmet mental health and oral health needs. Unfortunately, healthcare financing does not address the financial barriers resulting from the high costs of health services they face [10]. Poor policies and healthcare providers´ prejudices, attitude and sensitivity also create structural barriers that limit adolescents´ access to care. Although these barriers exist for adolescents in high-income settings, the impact in LMICs is more severe because of greater inadequacies in health systems, health financing, and the socio-cultural, economic and political environments [11].

The interconnectedness of adolescent OMSRH requires that healthcare professionals who address these needs work together. This cooperation requires using a strategic approach that can facilitate adolescents´ access to universal health coverage free of catastrophic financial cost. Integrated healthcare delivery to address these priorities is an appropriate strategy. Integrated healthcare is “the organization and management of health services so that people get the care they need, when they need it, in ways that are user-friendly, achieve the desired results and provide value for money” [12]. Integrated healthcare is congruent with universal health coverage and quality primary health care and is a viable approach to comprehensively addressing adolescent OMSRH. Integrated care also is consistent with the World Health Organization guidance on adolescent oral [13], mental [14] and SRH [15], especially in resource-limited settings.

Integration of adolescent health services could be especially useful in resource-limited countries with large economic and health needs, such as Nigeria. Nearly a quarter of Nigeria´s 2019 population of 202 million people are 10- to 19-year-old adolescents [16]. Additionally, at 107 per 1000, Nigeria has one of the highest global birth rates among adolescent girls 15 to 19 years of age [17]. Finally, reports indicate significant system-level gaps in oral [18], sexual/reproductive and mental health care [19] for children and adolescents in Nigeria. Using Nigeria as an example, this paper theorizes an integrated approach for adolescent OMSRH, using the oral health platform for improving access and uptake and, ultimately, outcomes. We propose a one-stop-shop (OSS) model to facilitate adolescents´ access to an integrated OMSRH care service in ways that addresses the growing demand for improved patients´ uptake of services, experience and health outcomes. The availability and appropriateness of health interventions in adolescence is likely to have long-term impact on health, including OMSRH. These interventions, combined with the interconnectedness of OMSRH needs, makes vertical and horizontal integration of care appropriate for adolescents. A vertically integrated healthcare system enables establishment of a broad range of patient care and support services to adolescents [20], including access to services outside of facilities. Horizontal care integration is the coordination of activities across the facility operating the OSS for OMSRH service [21].

**Oral health clinics as a non-stigmatized service delivery point:** the delivery of OMSRH services to adolescents must be free of stigma. Lessons on facilitating adolescents´ access and use of stigmatized services, such as SRH and mental health, can be learned from the years of delivering HIV care and support services to adolescents living with HIV. Stigmatized service for adolescents result in significant loss of clients. Oral health clinics offer non-stigmatized services in private, safe spaces for discussion of sensitive mental and SRH issues. Adolescents in Nigeria seeking SRH services are often concerned about breach of confidentiality [21], so training of healthcare providers to address these concerns within an OSS model will be necessary. Besides being less stigmatizing, oral health clinics have the potential to increase adolescents´ use of oral health services. The need for oral healthcare is often considered less urgent than the need for other services, such as those for mental and SRH. Adolescents in Nigeria use oral healthcare service mainly for urgent/emergent curative purposes, not necessarily because of financial challenges, but due to poor perception on the need for preventive oral care. The proposed integrated care delivery model has the potential to reverse this trend.

**Youth-friendly services:** acceptable youth-friendly services are accessible, equitable, appropriate and effective for various youth subpopulations, and help to overcome the challenges of traditional healthcare models. The services are supported by rights-based, youth-friendly policies, and the clinics are made comfortable and flexible to meet adolescents' needs OMSRH services should be delivered through youth-friendly facilities. Youth-friendly clinics are more accessible for young people than are the usual health-care services, which are plagued with sociocultural norms and policies that enforce age-related competency for informed consent, poor funding, weak linkages to other required services, and health systems that are unresponsive to adolescents´ complex and diverse health needs [22]. In the integrated model we describe, OMSRH service providers are trained to provide youth-friendly services. Training can improve competency of healthcare providers for youth-friendly services. Competency-based training on youth-friendly service is being conducted under multiple initiatives in Nigeria [23]. One of the challenges with training health personnel to provide quality care for preventive/chronic disease in Nigeria is the continual rotation of staff. In-service training for oral healthcare providers may be cost-effective for Nigeria as these providers are less likely to be rotated out of the oral health clinic because their specialized skills are often applicable only to oral health care. Dental clinics are more amenable to structural changes that can improve adolescents´ use of services for OMSRH. For example, the waiting areas of dental clinics can be made attractive for young people, and the clinics can provide services during evening hours.

**Figure 1:**
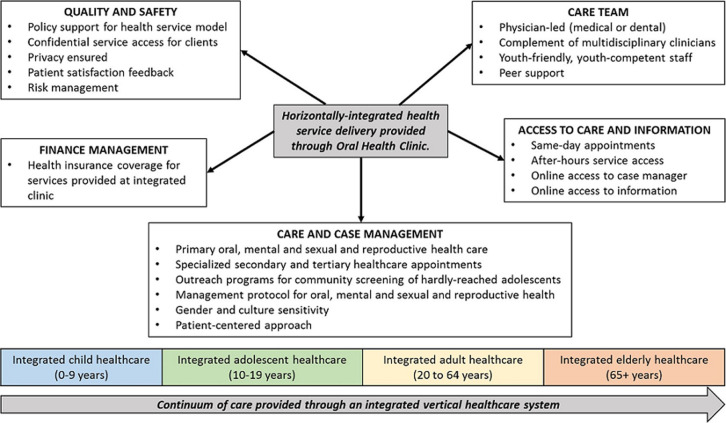
conceptual framework for integrated care access for adolescents along a continuum of care

**Facility-based services**: providing youth-friendly services within institutionalized structures promotes sustainability. Facility-based healthcare delivery for adolescents can promote integration of adolescents into a facility-based life-time continuum of care. Children can transition into the OSS adolescent OMSRH clinics to access preventive and therapeutic care, and adolescents can transition into adult healthcare clinics, thereby promoting vertical healthcare integration, as illustrated in Figure 1. Facility-based service promotes coordinated, comprehensive, people-centered, population-specific quality health care throughout life [24]. For LMICs like Nigeria, access to services through community-based service delivery points is more acceptable than facility-based service for adolescents: adolescents are less likely to access facility-based services in these settings [25]. Community-based youth-friendly services are more effective than facility-based interventions for delivering stigmatized care [26]. The OSS model will require systems and structures that are linked to community resources and can help meet adolescent OMSRH needs and empower the youths to manage their health and healthcare. The OSS service model is expected to mature into a model that facilitates differentiated care - a care approach that is tailored to intra-populations needs. Community structures for complementary services may include primary and secondary school-based clinics, although most Nigerian schools do not provide oral and mental health care. When such services are available in school clinics, their use is limited by concerns about confidentiality because of the close link to school authorities, peers, and parents. There is little documentation on the use of school clinics to provide oral, mental and SRH services to adolescents in LMICs [27].

**The one-stop-shop OMSRH service delivery model for adolescents:** we propose an OSS, integrated OMSRH model that promotes continuous access of adolescents to care and information, managed by a competent, youth-friendly, multidisciplinary team of healthcare providers led by a physician. The model will require institution-based financing. One mechanism proposed for the OSS is discussed below. Systems must also be in place for quality control and assurance and for safety enhancement. The use of shared guidelines and protocols by the healthcare team will facilitate coordinated OMSRH care, and the clinic´s linkage with community systems will be important for the adolescent´s development [1]. Table 1 provides details of the objectives and strategies of the OSS delivery model for adolescent OMSRH care. Figure 1 provides the details for the design of the system. The delivery of care will be a lifetime endeavor. The fulcrum is the oral health clinic, whose clinical services will be the template for integration of mental and SRH services for adolescents. The initial stages of integration will be within single, large facilities that have established oral health clinics and separate mental health and SRH service points. Subsequent phases of integration will involve facilities that have only one or two established clinics for oral, mental or SRH. To facilitate the prompt diagnosis of OMSRH conditions and attention to preventive care, staff will need to be trained on the use of the Home, Education, Activities, Drugs, Sexuality, Suicide/Depression, Safety and Strengths/Spirituality psychosocial tool [28]. Through this tool, adolescents with identified issues can have access to education, counseling and basic treatment and, where needed, referral for specialized care.

**Table 1:** key components and strategies for a one-stop-shop model to deliver adolescent oral, mental, sexual and reproductive health services

Domain	Objectives	Strategies
Health System	Create an integrated unit that promotes safe, high-quality care accessed through a facility-based service delivery point for adolescents	Develop agreements that facilitate care coordination within and across different levels of healthcare systems
		Develop robust referral mechanism between community structure and facility
		Encourage open and systematic handling of errors and quality problems to improve adolescent care
		Improve support of the management of all levels of care in the organization for the care concept
		Promote strategies aimed at comprehensive system change including development of referral mechanisms that support access of all adolescents to the one-stop-shop for screening services
		Provide healthcare provider incentives based on quality of care
Delivery System Design	Assure the delivery of effective, efficient clinical care and self-management support for adolescents	Develop integrated OMSRH care management protocol
		Ensure short waiting time for scheduled appointments
		Ensure access to real and virtual information
		Ensure access to preventive services like sexually transmitted infection screening and HPV immunization
		Ensure regular follow-up by the care team
		Facilitate access to existing support structures
		Promote gender sensitive care delivery services
		Promote quality and safety
		Provide finance support for service access
		Train care team to be youth-friendly, and competent to provide individualized patient-centered care
Self-Management Support	Empower and prepare adolescents to manage their health and health care	Emphasize patient's central role in managing their health
		Use effective self-management support strategies that include assessment, goal-setting, action planning, problem-solving and follow-up
		Use proven provider counselling and education methods to promote adolescents' interest in self-care
		Organize internal and community resources to provide ongoing self-management support
Clinical Information Systems	Organize patient and population data to facilitate efficient and effective care	Facilitate individual patient care planning
		Identify relevant subpopulations for proactive care
		Monitor performance of practice team and care system
		Provide timely reminders for patients
		Share information with patients and providers to coordinate care
Community Systems	Mobilize community resources to meet needs of adolescents	Advocate for policies to improve adolescent care
		Encourage patients to participate in peer support community-based programs
		Form partnerships with community organizations to support and develop interventions to fill service gaps
		Institute collaborative support for community structure by facility personnel

Adapted from: Curry N, Ham C. Clinical and service integration. The route to improved outcomes. The King's Fund. 2010. Available at: https://www.kingsfund.org.uk/sites/files/kf/Clinical-and-serviceintegration-Natasha-Curry-Chris-Ham-22-November-2010.pdf. Accessed 30 June, 2019

The OSS also needs to address the limited financial independence of adolescents. Adolescents´ out-of-pocket expenses for healthcare can be addressed through a non-profit health plan that bears insurance risks for beneficiaries who access these services. Kaiser Permanente´s healthcare financing approach [29] can be adapted, whereby the health institution/integrated OMSRH clinic receives financial capitation for OMSRH services delivered to adolescents. The national health insurance scheme in Nigeria can pay the capitation for service delivery. For youth friendly OMSRH care to function optimally, the capitation should be independent of parental acknowledgement of the service received. This approach can facilitate adolescents´ access to services independent of parental financial constraints, which is not the routine practice in Nigeria. The package of care for OMRSH for adolescents provided through the OSS will be in line with the package of care proposed for the level of health care delivery instituting the integrated in line with the oral health, mental health and SRH care package in Nigeria. We have proposed an OSS-OMSRH service delivery model aimed at providing quality health care for adolescents requiring OMSRH care. The approach is a potentially cost-effective strategy for managing health problems with common etiological risk factors and interconnected healthcare needs. It uses a common risk factor approach to contextualize OMSRH access by adolescents. The approach recognizes the inter-professional nature of integrated care and promotes professionals coming together to provide multi-faceted care through a single delivery point.

An integrated OMSRH management approach is expected to facilitate the diagnosing and management of OMSRH issues for a substantial proportion of Nigerian adolescents at high risk. There are currently no guidelines for the implementation of integrated oral, mental, sexual and reproductive health programs for adolescents, although there are policy documents that discuss oral, mental and SRH. Implementation of these policies, even separately, is lacking in many LMICs. Some reasons for this lack are the sociocultural norms and socio-economic issues that make adolescents dependent on parents for health access; less flexible response of health systems to health needs of youths; limited capacity of healthcare professionals to provide youth-friendly care; poor funding for adolescent health needs; and poor linkage of facility-based and public health services with the community and other sectors [30]. While operation of the OSS may be constrained by some or all the challenges listed above, they should not preclude initiation of structural change. First, piloting the OSS- OMSRH model would provide preliminary data to facilitate testing it in a rigorously designed implementation research study, providing evidence for adoption and scale-up. Second, where there is a recognized need for a convenient, less-stigmatizing, more attractive space from which to provide often-stigmatized mental and SRH services, this need can fast-track the prioritization of oral healthcare service within existing primary healthcare systems and structures [18]. However, the limited availability of dental clinics in LMICs like Nigeria may interfere with implementation of our proposed model for adolescent OMSRH care. Table 2 and Table 3 show the distribution of dental clinics by ownership in Nigeria and dental personnel serving the Nigerian population as 2013/2014.

**Table 2:** distribution of dental clinics in Nigeria, by operational ownership*

Owner	Geo-Political Zones						TOTAL N=679 n (%)
	South-West N=256 n (%)	North-Central N=136 n (%)	North-West N=110 n (%)	South-South N=90 n (%)	South-East N=53 n (%)	North-East N=34 n (%)	
Federal Government	17 (6.6)	30 (22.1)	17 (15.5)	11 (0.1)	6 (11.3)	7 (20.6)	88 (12.9)
State Government	56 (21.9)	41 (6.6)	50 (45.4)	24 (26.7)	9 (17.0)	20 (58.8)	200 (29.5)
Local Government	2 (0.8)	0 (0.0)	25 (22.7)	0 (0.0)	0 (0.0)	0 (0.0)	27 (3.9)
Private Organizations	177 (69.1)	61 (44.9)	17 (15.5)	51 (56.7)	35 (66.0)	7 (20.6)	348 (51.2)
Corporate Organizations	2 (0.8)	1 (0.7)	1 (0.9)	2 (2.2)	0 (0.0)	0 (0.0)	6 (0.9)
Mission Hospitals	1 (0.4)	3 (2.2)	0 (0.0)	1 (1.1)	3 (5.7)	0 (0.0)	8 (1.2)
Unspecified	1 (0.4)	0 (0.0)	0 (0.0)	1 (1.1)	0 (0.0)	0 (0.0)	2 (0.3)

The information is an extract from the report of the research on the survey of Oral Health Manpower, Facilities and Training Institutions in Nigeria, conducted by the Inter-country Centre for Oral Health (ICOH) for Africa, Jos 2013/2014 and submitted to the Federal Ministry of Health

**Table 3:** distribution of dental surgery specialists in Nigeria*

Specialty type	Geo-political zones						Total N =1,618 n (%)
	South-West N=775 n (%)	North-Central N=211 n (%)	North-West N=181 n (%)	South-South N=286 n (%)	South-East N=130 n (%)	North-East N=35 n (%)	
Oral and maxillofacial surgery	39 (23.9)	13 (59.1)	14 (66.7)	27 (36.5)	9 (37.5)	1 (50.0)	102 (33.3)
Community dentistry	15 (9.2)	0 (0.0)	1 (4.8)	6 (8.1)	2 (8.3)	0 (0.0)	24 (7.8)
Periodontology	9 (5.5)	1 (4.5)	0 (0.0)	11 (14.7)	2 (8.3)	0 (0.0)	23 (7.5)
Oral medicine	7 (4.3)	0 (0.0)	1 (4.8)	5 (6.8)	2 (8.3)	0 (0.0)	15 (4.9)
Oral radiology	0 (0.0)	0 (0.0)	0 (0.0)	0 (0.0)	0 (0.0)	0 (0.0)	0 (0.0)
Paedodontics	16 (9.8)	1 (4.5)	0 (0.0)	4 (5.4)	2 (8.3)	0 (0.0)	23 (7.5)
Orthodontics	25 (15.3)	2 (9.1)	1 (4.8)	11 (14.7)	2 (8.3)	0 (0.0)	41 (13.4)
Oral pathology	13 (8.0)	0 (0.0)	1 (4.8)	2 (2.7)	1 (4.2)	0 (0.0)	17 (5.6)
Conservative dentistry	29 (17.8)	4 (18.2)	2 (9.5)	5 (6.8)	3 (12.5)	1 (50.0)	45 (14.7)
Prosthodontics	10 (6.1)	1 (4.5)	1 (4.8)	3 (4.1)	1 (4.2)	0 (0.0)	16 (5.2)
Number of dentists with specialist training	163	22	21	74	24	2	306

The information is an extract from the report of the research on the survey of Oral Health Manpower, Facilities and Training Institutions in Nigeria, conducted by the Inter-country Centre for Oral Health (ICOH) for Africa, Jos 2013/2014 and submitted to the Federal Ministry of Health.

There are 679 dental clinics and 1,618 dental surgeons providing oral health care for the entire country of ~200 million people in Nigeria. Most (53.3%) of the dental clinics are operated by private/corporate/religious organizations; only 3.9% of public oral health facilities are operated from primary healthcare facilities at the local government level as shown in Table 1. More adolescents live in rural than in urban Nigeria; thus, the cost of travel to oral health clinics, which are concentrated in urban areas, may be a barrier to access. Financial barriers to service can be reduced through financial incentives, although there are objections because they may prompt change in behaviors to cash transaction. There are no accessible studies on the use of financial incentives for improving adolescents´ access to services. In LMICs, if the scope and scale of public oral health facilities expand, as primary healthcare services are scaled up, the need to travel distances to access care services will decrease. A major limitation of our proposed OSS-OMSRH model is that it is based on a narrative literature review. We may have missed pertinent, more objective information that a systematic review would have yielded, but such a review was not possible because of the paucity of empirical studies on integrated health services for adolescents.

In conclusion, integrated-care OSS promising models for delivering comprehensive, accessible care to adolescents in a less-stigmatizing setting. These models may be applicable to other forms of stigmatized services in similar resource-limited settings. However, little data are available on use of these integrated services for adolescents, and even less for services integrated across vastly different health areas. Our proposed leveraging of dental clinics for integrated OMSRH services theorizes the provision of facility-based services to adolescents, with emphasis on reducing stigma as a barrier to service accessibility, acceptability, equitability and appropriateness. Empirical studies will be required to test the feasibility, validity and effectiveness of this proposed model.
